# Marker assisted pyramiding of drought yield QTLs into a popular Malaysian rice cultivar, MR219

**DOI:** 10.1186/s12863-016-0334-0

**Published:** 2016-01-27

**Authors:** Noraziyah Abd Aziz Shamsudin, B. P. Mallikarjuna Swamy, Wickneswari Ratnam, Ma. Teressa Sta. Cruz, Anitha Raman, Arvind Kumar

**Affiliations:** Faculty of Science and Technology, Universiti Kebangsaan Malaysia, 43600 Bangi, Selangor Malaysia; International Rice Research Institute, Los Banos, DAPO BOX 7777 Metro Manila Philippines

**Keywords:** Drought tolerance, Marker assisted breeding, *qDTYs*, QTLs pyramiding

## Abstract

**Background:**

Three drought yield QTLs, *qDTY*_2.2_, *qDTY*_*3.1*,_ and *qDTY*_*12.1*_ with consistent effect on grain yield under reproductive stage drought stress were pyramided through marker assisted breeding with the objective of improving the grain yield of the elite Malaysian rice cultivar MR219 under reproductive stage drought stress. Foreground selection using QTL specific markers, recombinant selection using flanking markers, and background selection were performed. BC_1_F_3_-derived lines with different combinations of *qDTY*_*2.2*_, *qDTY*_*3.1*,_ and *qDTY*_*12.1*_ were evaluated under both reproductive stage drought stress and non-stress during the dry seasons of 2013 and 2014 at IRRI.

**Results:**

The grain yield reductions in the stress trials compared to non-stress trials ranged from 79 to 93 %. In the stress trials, delay in days to flowering and reduction in plant height were observed. In both seasons, MR219 did not produce any yield under stress, however it produced a yield of 5917 kg ha^−1^ during the 2013 dry season and 8319 kg ha^−1^ during the 2014 dry season under non-stress. Selected introgressed lines gave a yield advantage of 903 to 2500 kg ha^−1^ over MR219 under reproductive stage drought stress and a yield of more than 6900 kg ha^−1^ under non-stress during the 2014 dry season. Among lines with single *qDTY*, lines carrying *qDTY*_*2.2*_ provided a higher yield advantage under reproductive stage drought stress in the MR219 background. Two-*qDTY* combinations (*qDTY*_*3.1*+_*qDTY*_*2.2*_ and *qDTY*_*3.1*+_*qDTY*_*12.1*_) performed better than lines with three *qDTY* combinations, indicating the absence of positive interactions between the three *qDTYs*.

**Conclusion:**

We successfully developed drought-tolerant MR219 pyramided lines with a yield advantage of more than 1500 kg ha^−1^. Differential yield advantages of different combinations of the *qDTYs* indicate a differential synergistic relationship among *qDTYs*. This is the first report on the successful effect of *qDTYs* in increasing the yield under drought in genetic backgrounds other than those in which the *qDTYs* were earlier identified.

## Background

Drought is the major constraint to rice production and yield stability in rainfed rice-growing areas in many Asian countries. The severity of drought varies with rainfall pattern, irrigation source, soil type, water availability within and between seasons, and stage of crop growth [1], causing the varied responses of rice cultivars in different years and environments. In South and Southeast Asia as well as in Africa, severe drought is observed almost every year, which drastically affects rice production [2]. In Asia alone, about 45 % of the total rice-growing areas have no assured irrigation access and are subjected to frequent drought [3]. Drought adversely affects the rice crop at all stages of growth and early reproductive stage drought stress (RS), especially during anthesis, has been found to result in significant yield reduction as also observed in wheat and barley [4, 5]. The reduction in rice yield is frequently associated with the increased percentage of spikelet sterility [6–8] and spikelet number per panicle [9]. The degree of seed yield reduction due to water deficit is highly dependent on the timing and duration of stress [10]. Also, water deficit at the meiotic stage has been reported to reduce the seed set in some cultivated rice varieties [11]. The ability of the rice crop to withstand dry conditions and to reproduce in limited water conditions is essential for rice production to still prosper despite drought [12, 13]. It is, therefore, vital to focus on the development of high yielding drought-tolerant rice cultivars which have a targeted yield advantage of at least 1000 kg ha^−1^ over popular and widely adapted varieties under drought. However, breeding efforts for drought-tolerant rice varieties are limited due to factors such as the difficulty of defining a representative RS condition as well as the low heritability (*H*) of yield component traits such as spikelet sterility, relative water content, root pulling force, root dry weight, and harvest index under RS as these are highly influenced by multiple genes, the environment, and the interrelation between genotype and environment as well as interaction with other abiotic and biotic stresses [14].

Marker assisted breeding (MAB) has provided new opportunities to introgress regions governing tolerance to RS in drought-tolerant donors through careful QTL identification and fine mapping studies. At the International Rice Research Institute (IRRI), traditional and improved donors were used in developing mapping populations for the identification of major *qDTYs* [15]. As a result, several *qDTYs* with large and consistent effects such as *qDTY*_*1.1*_ [16, 17], *qDTY*_*2.1*_ [18]*, qDTY*_*2.2*_ [19, 20]*, qDTY*_*3.1*_ [18]*, qDTY*_*4.1*_ [20]*, qDTY*_*6.1*_ [21]*, qDTY*_*9.1*_ [20]*, qDTY*_*10.1*_ [20], and *qDTY*_*12.1*_ [22] were identified. Generally, these major effect *qDTYs* have a genetic gain of 10 to 30 %, with a yield advantage of 150 to 500 kg ha^−1^ under RS. However, to provide more significant economic benefits to farmers, a yield advantage of at least 1000 kg ha^−1^ is required [1]. In the past few years, consistent efforts have been made to introgress the identified *qDTYs* into drought-susceptible mega-varieties through the MAB strategy.

Rice breeding programs in Malaysia have focused on developing high productivity rice varieties and have come up with many high-yielding cultivars such as MR84, MR219, and MR220. Most of these cultivars are susceptible to drought. Several studies on the genetic diversity and the morphological, biochemical, and physiological responses of Malaysian rice germplasms under controlled drought environments have been conducted. However, not many studies were undertaken to improve the yield of current popular varieties under drought or to develop new drought-tolerant rice genotypes. Swamy et al. [20] reported that introgression lines with two and three *qDTYs* in an IR64 background gave a yield advantage of 1200 to 2000 kg ha^−1^ under RS as well as yields that were similar to that of IR64 under non-stress (NS) conditions, yet the effect of the identified *qDTYs* in diverse genetic backgrounds remains unknown. Thus, in this study, three drought-tolerant improved lines developed at IRRI and which have performed well under NS conditions were used as *qDTY* donors. IR 84984-83-15-18-B is the donor of *qDTY*_*12.1*_, the only *qDTY* for upland environment used in this study. This line was derived from a cross between Way Rarem, a high-yielding drought-sensitive Indonesian upland rice cultivar, and Vandana, a high-yielding and drought-tolerant Indian upland rice cultivar. Way Rarem belongs to the *indica* group while Vandana has 50 % *japonica* and 50 % *aus* background. The *qDTY*_*12.1*_ QTL was flanked between RM28048 and RM511 on chromosome 12. This QTL explained about 51 % of the total genetic variance with an estimated additive effect of 172 kg ha^−1^ for yield observed under severe upland RS over two years of field evaluation at IRRI [23]. IR 77298-14-1-2-10 and IR 81896-B-B-195 are the donors of *qDTY*_*2.2*_ and *qDTY*_*3.1*_, respectively, which were both identified under severe lowland RS condition. IR 77298-14-1-2-10 was derived from the cross between two *indica* varieties: AdaySel, a drought-tolerant Indian rice cultivar, and IR64, a modern cultivar grown in South Asia which is highly susceptible to RS. *qDTY*_*2.2*_ was flanked between RM109 and RM279 on chromosome 2 and explained 33 % of the genetic variance under severe lowland RS [18]. IR 81896-B-B-195 was derived from a cross between Apo, an improved *indica* upland variety with high yield potential under aerobic condition, and Swarna, a widely grown *indica* rainfed lowland Indian rice cultivar [18, 24]. *qDTY*_*3.1*_ was flanked between RM520 and RM16030 on chromosome 3, explaining about 31 % of the genetic variance for the trait [18]. In the present study, the three *qDTYs* were pyramided through stepwise marker assisted QTL pyramiding into the high-yielding Malaysian rice cultivar MR219 with the objectives of (i) improving its yield under drought, (ii) understanding the effect of different QTLs in enhancing yield in the background of MR219 individually and in combinations, and (iii) gaining a better understanding of QTL interactions to obtain higher yield advantage under drought.

## Result

### Development of BC_1_F_3_ pyramided lines using marker assisted breeding

**Fig. 1:**
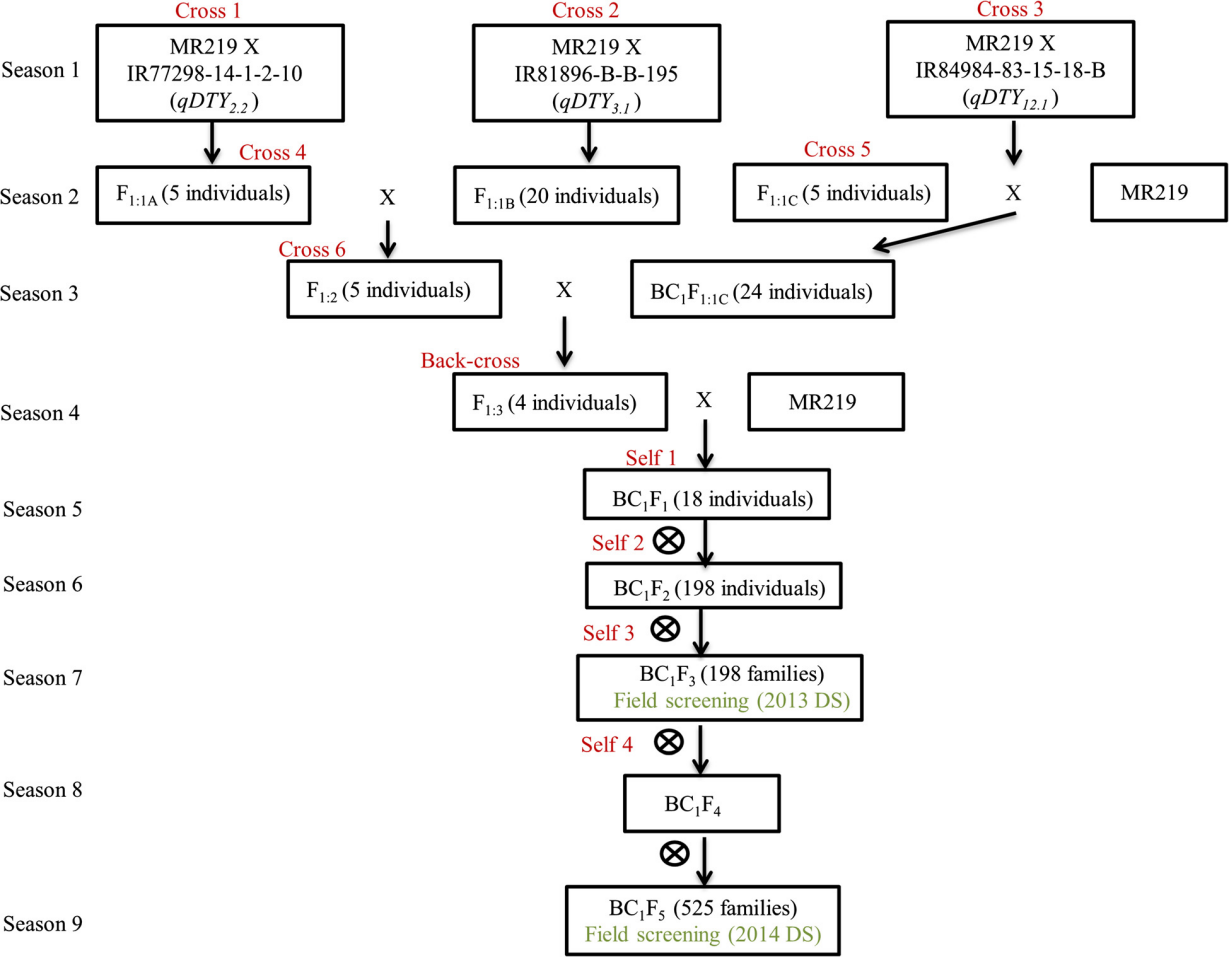
Crossing scheme for the development of BC_1_F_5_ MR219 pyramided lines and the number of plants selected at every generation

The number of selected individuals in every generation of BC_1_F_3_ development is shown in Fig. 1. In the 1st season, 96 % of the total F_1:1A_ individuals from Cross 1, 94 % of the total F_1:1B_ individuals from Cross 2, and 96 % of the total F_1:1C_ individuals from Cross 3 amplified the alleles of both parents (heterozygous). This indicated their true hybrid nature as confirmed using peak simple sequence repeat (SSR) markers at each *qDTY* locus (RM236 for *qDTY*_*2.2*_, RM520 for *qDTY*_*3.1*,_, and RM511 for *qDTY*_*12.1*_). In the 2nd season, Cross 4 was made to develop the F_1(2)_ population by crossing five confirmed F_1:1A_ individuals with 20 confirmed F_1:1B_ individuals. From 587 F_1(2)_ individuals genotyped, only 14 individuals showed donor alleles at both the *qDTY*_*2.2*_ and *qDTY*_*3.1*_ loci when ran with the peak and foreground SSR markers of these loci (OSR17, RM236, RM12460, RM279, RM12569, RM12949, RM12992, RM520, RM416, and DTY3-14). At the same time, 141 BC_1_F_1:1C_ individuals from Cross 5 were also genotyped for the presence of the *qDTY*_*12.1*_ locus using six peak and foreground SSR markers (RM28076, RM28099, RM28130, RM511, RM1261, and RM28166). However, only 24 BC_1_F_1:1C_ individuals were amplified *qDTY*_*12.1*_ alleles.

In the 3rd season, Cross 6 was made to produce the F_1(3)_ population by crossing 24 BC_1_F_1:1C_ selected individuals with the five F_1(2)_ individuals (IR 97992, IR 97995, IR 97998, IR 98001, and IR 98003)_*.*_ These five F_1(2)_ individuals were selected from the 14 F_1(2)_ individuals with two *qDTYs* (*qDTY*_*2.2*_ + *qDTY*_*3.1*_) as they had morphological characteristics similar to that of MR219. In the 4th season, a total of 472 F_1(3)_ individuals were genotyped for the presence of all the three *qDTY* loci and 24 F_1(3)_ individuals were amplified specific alleles of all the three *qDTYs*. However, only four F_1(3)_ individuals which had similar morphological characteristics as that of the recipient parent and with the higher recipient parent genomes (83 % in IR 98001-7, 89 % in IR 98003-130 and IR 98003-58, and 94 % in IR 98003-257) were further backcrossed with MR219 to produce the BC_1_F_1_ population.

In the 5th season, from the total of 1263 BC_1_F_1_ individuals genotyped, 104 individuals were amplified specific alleles of all the three *qDTYs*. However, only 18 BC_1_F_1_ individuals which had similar morphological characteristics to that of MR219 were selected. These 18 selected BC_1_F_1_ individuals were then genotyped with 48 background SSR markers, results of which showed that the genome recovery varied from 83 % in IR99778-60 to 99 % in IR99784-4. Thus, these 18 selected BC_1_F_1_ individuals were selfed to generate a large number of BC_1_F_2_ seeds. In the 6th season, a total of 5677 BC_1_F_2_ individuals obtained from the 18 individuals from the BC_1_F_1_ populations were genotyped for the presence of specific alleles of all the three *qDTY* loci, and results showed that 437 BC_1_F_2_ individuals were homozygotes at the different *qDTY* loci and their combinations. Furthermore, only 33 BC_1_F_2_ individuals carried all the three *qDTYs*. From a total of 437 BC_1_F_2_ individuals that were homozygotes at different *qDTY* and their combinations, only 198 BC_1_F_2_ individuals were finally selected based on their morphological similarity to MR219 and were further advanced in the 7th season to develop 198 BC_1_F_3_ families of pyramided lines (PLs).

### Imposition of drought stress

**Fig. 2:**
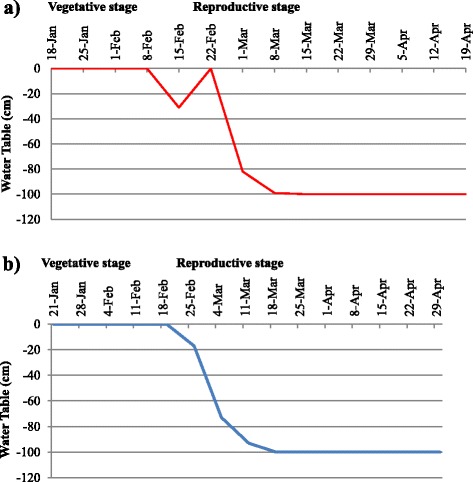
Parching water table in stress trials; **a** during 2013 dry season; **b** during 2014 dry season

**Fig. 3:**
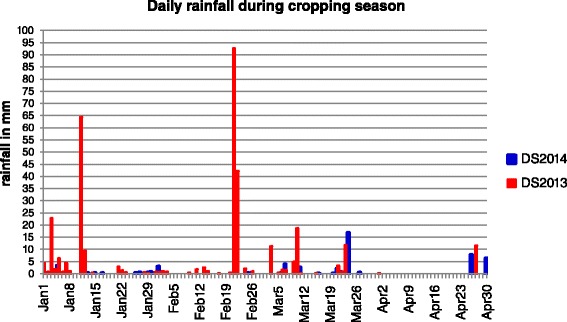
Daily rainfall during the dry season experiment period from January to April in 2013 and 2014

**Fig. 4:**
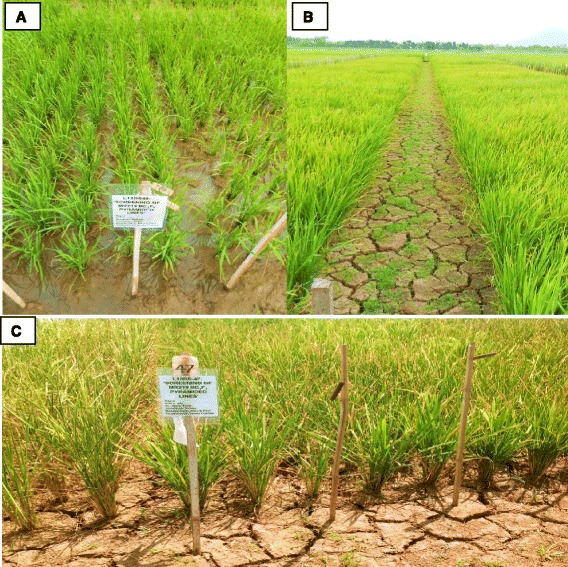
Crop at various stages after stress imposition **a** after 3 weeks of transplanting; **b** at reproductive stage; **c** at severe stress showing leaf rolling

Water table depth in the experimental plots of the RS trials during the dry season (DS) of 2013 and 2014 are shown in Fig. 2. Daily rainfall data at the IRRI experimental field during the months of January to April in 2013 DS and 2014 DS were also taken (Fig. 3). In the 2013 DS, the total rainfall was 257.7 mm and the RS treatment was initiated in the 2nd week of February while the stress trial was not irrigated until February 12. However, the stress trials received 137.6 and 37.8 mm rainfall in the 3rd week of February and 1st week of March, respectively, and only 0 to 15.5 mm rainfall from then on up to the last week of April. In the 2014 DS, the RS treatment was initiated also in the 2nd week of February and the stress trial received only 43 mm total rainfall from the day the stress was imposed until harvest. Ground water table continued to decrease to up to 100 cm within a month until harvest. Furthermore, the average water table depth during the critical flowering stage was 100 cm in both seasons, indicating that the crop faced severe RS in both seasons. Figure 4 shows the rice crop in its various growth stages in the RS trials after stress imposition.

### Validation of marker assisted breeding for drought tolerance by phenotyping

#### Line means and heritability

The overall performance of the MR219 PLs and their recipient parent is shown in Table 2. Mean days to 50 % flowering (DTF) in the MR219 PLs varied from 81 to 99 days in the NS trials and 87 to 92 days in RS. Flowering was delayed by 6 to 9 days in the RS trials as compared to the NS trials. Plant height (PH) ranged from 60 to 71 cm in the RS trials and from 87 to 95 cm in the NS trials. RS reduced PH by 24 to 30 cm. The mean grain yield (GY) of PLs ranged from 397 to 920 kg ha^−1^ in the RS trials and from 6040 to 7120 kg ha^−1^ in the NS trials. The 86 to 93 % reduction in yield showed that the MR219 PLs were subjected to severe RS in both 2013 DS and 2014 DS. The H of DTF was high in both NS and RS trials (Table 2). For PH, the H value was low to moderate in the RS trials but moderate to high in the NS trials. The H of GY was medium to high in the RS trials and medium in the NS trials.

### Performance of promising drought tolerant pyramided lines

The yield performance of 16 most promising drought-tolerant BC_1_F_5_ MR219 PLs is presented in Table 3. In the 2013 DS and 2014 DS, the recipient parent, MR219, did not flower in the RS trials. The differences in the DTF of the MR219 PLs under NS and RS conditions were considerable (86 to 94 days under NS and 92 to 105 days under RS). In the NS trials, the DTF for the chosen MR219 PLs was relatively lower than that of MR219 (data not shown). Differences in PH for the chosen MR219 PLs under RS and NS were also notable (82 to 101 under NS and 55 to 69 cm under RS). Mean PH for the selected MR219 PLs in both RS and NS trials was lower than that of MR219. The GY of selected MR219 PLs ranged from 6947 to 11,672 kg ha^−1^ in the NS trials and from 903 to 2523 kg ha^−1^ in the RS trials. MR219 produced very little or no GY under RS. However, under NS, the mean GY of MR219 ranged from 5917 to 8319 kg ha^−1^. Thus, the yield advantage of the PLs over MR219 under RS ranged from 756 to 2521 kg ha^−1^ in the 2013 DS and from 923 to 2523 kg ha^−1^ in the 2014 DS. In NS, most of these lines yielded similar to MR219 while some lines recorded higher yields than MR219 (Table 3).

### Performance of different combinations of *qDTY*

**Fig. 5:**
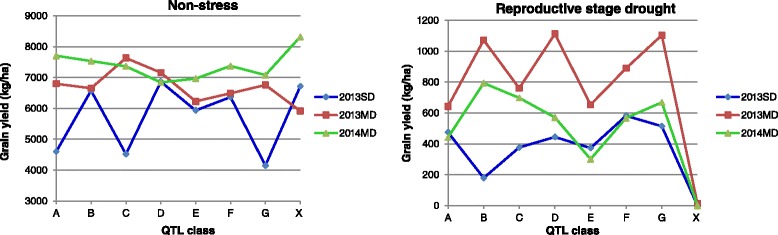
Graph showing QTL classes (*X axis*) and mean grain yield (*Y axis*) of short (SD) and medium duration (MD) lines with MR219 as the recipient parent. Trials were conducted during dry seasons of 2013 and 2014

The mean GY of the MR219 PLs with single and different combinations of *qDTYs* (QTL class – A, B, C, D, E, F, and G) alongside the recipient parent (no QTL class) is presented in Table 4. Under RS, the mean GY for the PLs was significantly higher than that of MR219. Generally, among MR219 PLs with a single *qDTY*, the mean GY of Class G (with *qDTY*_*2.2*_) was highest, followed by Class F (with *qDTY*_*3.1*_) and Class E (with *qDTY*_*12.1*_)_._ On the other hand, among the PLs with two *qDTYs,* Class E (*qDTY*_*2.2*_ *+ qDTY*_*3.1*_) followed by Class C (*qDTY*_*12.1*_ *+ qDTY*_*3.1*_) and Class B (*qDTY*_*12.1*_ *+ qDTY*_*2.2*_) provided a significant yield advantage over MR219 under RS. However, under NS, the yield levels of the recipient parent were higher compared to other QTL classes. These results indicate that PLs with *qDTY/s* were quite effective in enhancing GY under severe RS conditions (Fig. 5).

## Discussion

### Selection of parents

Developing and improving rice varieties with high GY under RS through different breeding strategies is necessary for obtaining sustainable rice yields even as the frequency and severity of drought are predicted to increase. However, like in all breeding programmes to develop superior lines, the selection of parents is a critical step. Until now, only one study has reported on improving drought tolerance in the Malaysian mega-variety MR219 which is known to be highly sensitive to drought [25]. However, the drought stress level used in the study was mild as the yield reduction of improved lines in the RS trials was less than 20 % compared to the control. According to [26], severe levels of RS with more than 65 % yield reduction in RS trials compared to control are necessary to identify true drought-tolerant lines.

Despite having a high adaptability to drought, several Malaysian traditional rice varieties and landraces usually carry undesirable traits such as low yield potential, poor response to high-input management, and taller plant type. One or more of these traits located close to *qDTY* regions, thus becoming a potential linkage drag in breeding for drought tolerance using conventional approaches. Moreover, in quantitative traits such as GY and PH, variability might be controlled by many minor QTLs. Thus, introgression of several minor genes to improve one quantitative trait is difficult to achieve due to two reasons: (i) the effect of minor genes is regularly inconsistent and (ii) there will be too many markers to handle in the breeding program. Different QTLs segregating in different mapping populations, QTL x genetic background interaction, and QTL x environment interaction could be the reasons for the inconsistency in the estimated QTL effects [27]. Thus, the selection of major effect QTLs to be used in breeding programs is essential. *qDTY*_*12.1*_ has shown the largest effect on GY under upland RS conditions while *qDTY*_*2.2*_ and *qDTY*_*3.1*_ have shown the larger effect on GY under lowland RS conditions [18, 22, 28]. It is interesting to note that these three *qDTYs* were identified in severe levels of RS under field conditions. Furthermore, *qDTY*_*2.2*_*, qDTY*_*3.1*,_ and *qDTY*_*12.1*_ also exhibited a consistent effect in different mapping populations. The use of pre-breeding lines or near isogenic lines with major effect QTL is the key to be successful QTL pyramiding [1, 29].

Pre-breeding lines that possess drought yield QTLs coupled with appropriate plant type and high yield have been generated at IRRI. These lines were produced by crossing low-yielding but drought-tolerant donors with high-yielding but drought-susceptible recipients [1]. In this study, MR219 was used as recipient parent as it carries desirable traits such as high yield potential, appropriate PH, and moderate to high tolerance to multiple pests and diseases. Pre-breeding lines in the background of high-yielding mega-varieties with a major and consistent effect *qDTY*, namely, IR 77298-14-1-2-10, IR 81896-B-B-195, and IR 84984-83-15-18-B were used as donor parents.

### Development of drought tolerant MR219 PLs using stepwise MAB technique

MAB has been an effective and efficient strategy in crop improvement as it speeds up and simplifies the selection process especially for complex traits [30–32]. Several researchers successfully pyramided QTLs/genes for multiple disease resistance to provide a broader spectrum of resistance than those conferred by a single QTL/gene [33–36].

Swamy and Kumar [1] reported that Vandana-introgressed lines with *qDTY*_*12.1*_ showed a yield advantage of only 500 kg ha^−1^ over the drought-tolerant cultivar Vandana. Thus, introgression of only a single *qDTY* into drought-susceptible mega-varieties may not produce adequate yield increments under RS conditions. In this study, several MR219 PLs gave 1000 kg ha^−1^ or more yield in the RS trials. This result indicates that marker-assisted QTL pyramiding of major-effect *qDTYs* through backcross breeding is an appropriate strategy to achieve an increase of at least 1000 kg ha^−1^ of GY under RS conditions.

Marker assisted pyramiding can also enable breeders to introgress two or more QTLs controlling various traits associated with biotic and abiotic stresses in plants. Frisch and Melchinger [37] concluded that the effectiveness of marker assisted breeding/pyramiding depends on the availability of closely linked markers and/or flanking markers for the target locus, the size of the population as well as the number of backcrosses, and the position and number of background markers. However, another study [38] indicated that the selection of the recipient and donor parents was more crucial. MAB with a stepwise screening technique was applied to select genotypes with desirable genes/QTLs by reducing a number of selected individuals in each step [35, 39].

In the present study, peak markers tightly-linked to the QTL region were selected. The linkage drag was eliminated using flanking markers while background markers were used for the fast recovery of the recipient parent’s genetic background [40]. Donor fragments of approximately 15 Mbp of the three drought yield QTLs, *qDTY*_*2.2*_ (10 Mbp)*, qDTY*_*3.1*_ (1.7 Mbp), and *qDTY*_*12.1*_ (3.5 Mbp) were introgressed into MR219 crosses. The PLs in which all the *qDTYs* were successfully introgressed represented about 3.8 % of the genome. With this size of the introgressed fragments, linkage drag might have occurred and affected the phenotype of the plants with *qDTYs*. This is partly evident from the variability in the DTF of the MR219 PLs as compared to the recipient parent. Dixit et al. [41] reported that the *qDTY*_*2.2*_ and *qDTY*_*12.1*_ regions were narrowed down, thus, introgression of these refined QTL regions could minimize the undesirable linkage of the *qDTY* donors.

The probability of obtaining at least one ideotype carrying homozygous loci in the F_2_ population is 4^n^, where ‘n’ is the number of target loci [42]. In the current study though, only 14 (F_1:2_) individuals with two *qDTYs* (*qDTY*_*2.2*_ + *qDTY*_*3.1*_) were identified from the total of 587 individuals produced in Cross 6. This indicates that the ideotype plant was absent probably because the crossing program involved four different genetic backgrounds. Therefore, a greater number of the F_1:2_ population is required to get one ideotype. Similarly in BC_1_F_2_ populations, only 33 individuals from the total of 5677 individuals with all three *qDTYs* in homozygous loci were identified, which confirmed that at least a doubled population size is required to get one desired ideotype. The same observation was reported by [35]. As the QTL regions were large, more than one marker for each QTL was used in step-wise system for the foreground selection. The larger size of the individual QTL regions as well as the necessity to pyramid two or more QTLs to obtain a sufficient number of positive plants with combinations of *qDTYs* requires maintaining a larger population than pyramiding genes/QTLs having tightly linked markers.

Introgression of QTLs involved in stress tolerance often induces undesirable traits from the donor parents. This might be due to the lack of a precise knowledge of the key genes underlying the QTLs controlling the target traits [38]. Servin and Hospital [43] reported that two to four markers on a chromosome of about 100 cM distance could provide adequate coverage of the genome on backcross programs through MAB simulation study. In this study, at least four markers per chromosome with an average distance of 35 cM between markers were used for background selection in the BC_1_F_1_ populations as recommended by [39, 40]. The selected BC_1_F_1_ MR219 PLs had 83 to 99 % of the recipient genome.

### Severity of reproductive stage drought stress trials

The level of drought in the 2013 DS and 2014 DS was considered as severe based on the mean yield reduction of more than 65 % as compared to NS (Table 2). The soil showed a deep crack due to insufficient water moisture (Figs. 2 and 4). In a different study, [44] reported yield reductions of up to 70 % upon imposing drought for 15 days at panicle initiation stage and 88 and 52 % reduction when stress was introduced at flowering and grain filling stage, respectively. Although [45] proposed that 50 % reduction in yield is required to identify true drought-tolerant lines, [46] did not, however, observe any response to selection in screens that had a yield reduction of up to 56 %. They recommended that a screening protocol that could reduce the mean yield by at least 65 % under RS as compared to irrigated control is required in order to identify true drought-tolerant lines.

### Agronomic performance of MR219 PLs under RS and NS conditions

Delay in flowering that was observed in the RS trials in this study confirmed that water stress affected flowering time. Similar results were reported by [16, 17, 19, 47–53].

MR219 was extremely sensitive to severe RS conditions as it did not flower under RS. However, MR219 PLs were less affected by RS probably due to the QTL alleles that exhibited a tendency to reduce delay in flowering under stress conditions. Similar results were observed in NS trials where MR219 PLs flowered earlier than their recipient parent. Bernier et al. [54] and Venuprasad et al. [19] showed that *qDTY*_*12.1*_ and *qDTY*_*3.1*_ affected both GY and DTF under RS conditions, suggesting that genes within these QTLs are probably associated with early DTF. In this study, stress was imposed 30 days after transplanting to ensure that even lines with earliest flowering did not escape drought and that the selected lines are truly drought tolerant.

### Performance of identified improved drought tolerance PLs

Sixteen BC_1_F_5_ MR219 PLs with yield advantages of 903 to 2523 kg ha^−1^ under RS during the 2014 DS were identified. The selected MR219 PLs also performed well in the NS trials. Two PLs, IR 99784-156-137-1-1 and IR 99784-188-201-B-1, gave yield advantages of 1042 kg ha^−1^ and 2523 kg ha^−1^, respectively, under severe RS and 1653 kg ha^−1^ and 2391 kg ha^−1^ yield advantages over MR219 under NS conditions. Comparison among the chosen PLs showed that 75 % and 63 % of them carried *qDTY*_*3.1*_ and *qDTY*_*2.2*_ either as a single *qDTY* or a combination with other *qDTYs.* PLs with *qDTY*_*12.1*_ either singly or in combination with other *qDTYs* was the lowest (56 %). However, no definite pattern could be assessed as to the performance of selected lines under RS due to the interaction between QTLs and background genotyping. This implies that QTL pyramiding using MAB technology is an effective method to improve current mega-varieties and to develop new rice cultivars that are tolerant to drought. PLs with good yield potential and an appreciable yield under RS can be effectively disseminated for cultivation by rice farmers in drought-prone environments of Malaysia.

### Performance of different combinations of *qDTY*

The higher mean GY of MR219 PLs compared to the recipient parent MR219 under RS is an indication of the positive effect of introgressed *qDTYs* under these conditions. Mean comparisons of *qDTY* classes show that introgressed lines with a single *qDTY* provided a significant yield increase over MR219 under RS without any yield reduction in NS, indicating the positive effect of *qDTY*_*2.1*_*, qDTY*_*3.1*_*,* and *qDTY*_*12.1*_ in the MR219 background to increase yield under RS. These QTLs have been found to show some effect in the background of the high-yielding varieties IR 64 [20], Swarna, and TDK 1 [19, 55]. This is the first report of introgression and yield increase produced by *qDTY*_*2.1*_*, qDTY*_*3.1*_*,* and *qDTY*_*12.1*_ in genetic backgrounds other than varieties against which the individual *qDTYs* were identified.

Mean comparisons of *qDTY* classes also show that introgressed lines with two *qDTYs* (*qDTY*_*2.2*_ *+ qDTY*_*3.1*_*, qDTY*_*2.2*_ *+ qDTY*_*12.1*_*,* and *qDTY*_*3.1*_ *+ qDTY*_*12.1*_) provided a significant yield advantage over MR219 under RS. However, the yield advantage provided by combining three *qDTYs* (*qDTY*_*2.2*_ *+ qDTY*_*3.1*_ *+ qDTY*_*12.1*_) was lower than the two-*qDTY* combinations. This demonstrates the absence of a positive interaction between three *qDTYs* even though each shows a positive interaction with each other. Mean comparisons of *qDTY* classes further reveal the role of interaction between *qDTYs* and between *qDTYs* and recipient genetic background. The results indicate: (i) a non-linear interaction between multiple *qDTYs* and (ii) the presence of a differential synergistic relationship between *qDTY* combinations [20, 56]. The *qDTY* combinations that provide higher yield advantage under RS may vary from genotype to genotype due to *qDTY* x *qDTY* and *qDTY* x genetic background interactions. The results imply the necessity to identify *qDTY* combinations with positive interaction against different genetic backgrounds for their precise use in MAB.

## Conclusion

Drought is a major challenge in achieving sustainable world rice production in the rainfed ecosystems of Asia. Breeding drought-tolerant rice cultivars can increase rice production yields especially in rainfed ecosystems under drought stress. The identification and introgression of QTL regions with a large and consistent effect on GY under RS presents an opportunity to improve high-yielding drought-susceptible mega-varieties through MAB. The selected MR219 PLs developed in this study conferred a yield advantage of 903 to 2523 kg ha^−1^ over their recipient parent under RS conditions and maintained high yield potential similar to or higher than MR219 under NS, indicating that drought tolerance can be successfully combined with high yield potential in the background of semi-dwarf varieties.

## Methods

### Plant materials and breeding scheme for development of BC_1_F_5_ population

All the plant materials including the mapping populations and PLs used in the study were developed at IRRI, Philippines. A total of 198 BC_1_F_3_ and 525 BC_1_F_5_ PLs were used in this study and were derived from the crossing of the drought-susceptible Malaysian rice cultivar MR219 (recurrent parent) with three drought-tolerant donor parents, namely IR 77298-14-1-2-10, IR 81896-B-B-195, and IR 84984-83-15-18-B. MR219 is a high-yielding indica rice cultivar with desirable traits such as short maturity, appropriate plant height with strong culms, and resistance to blast and bacterial leaf blight, while the grain can be marketed as a long-grain variety [57]. However, it is highly susceptible to drought. The donor parents, meanwhile, are the pre-breeding lines developed from mapping populations generated for QTL identification study by crossing drought-susceptible mega-varieties with drought-tolerant donors. Donor parents (Table 1) also carried desirable traits such as high yield, appropriate plant height, and medium to high resistance to pests and diseases as they were in the background of mega-varieties.

**Table 1:** Details on drought yield QTLs (*qDTYs*) used in the study

Recipient	Donor	Donor line used	Ecosystem	*qDTY* name	Chromosome	Interval	Peak marker	a	R^2^
IR64	Adaysel	IR77298-14-1-2-10	Lowland	*qDTY*_*2.2*_	2	RM236-RM279	RM236	14	6
Swarna	Apo	IR81896-B-B-195	Lowland	*qDTY*_*3.1*_	3	RM520-RM16030	RM520	30	27
Vandana	Way Rarem	IR84984-83-15-18-B	Upland	*qDTY*_*12.1*_	12	RM28048-RM511	RM511	47	33

Additive effect compared to trial mean (a, in percent), phenotypic variance (R^2^, in percent)

The three *qDTYs* were introgressed into MR219 using the QTL pyramiding technique as suggested by [39] and [35] which involved six crosses, followed by a backcross and four times of selfing thereafter (Fig. 1).

### Genotyping and marker assisted breeding

The DNA marking work was conducted at the Molecular Marker Applications Laboratory (MMAL) of the Plant Breeding, Genetics, and Biotechnology Division of IRRI. Fresh leaves from all lines were collected and freeze-dried. DNA extraction of leaf samples was carried out using the modified CTAB protocol [58]. A total of 125 SSR markers linked to the three *qDTY* regions (foreground selection) and an additional 711 SSR markers distributed in the whole rice genome which are unlinked to the *qDTY* regions (background selection) were tested in a polymorphism survey. However, only three peak markers and additional 13 flanking markers were found to be polymorphic in the three *qDTY* regions and were used in foreground selection in every generation. The peak markers linked to the three *qDTY* regions on chromosomes 2, 3, and 12 were RM236, RM520, and RM511, respectively (Table 1). All SSR markers were assayed on the MR219 rice population as described by [59]. The polymerase chain reaction products were separated in 6 % or 8 % non-denaturing polyacrylamide gel electrophoresis. DNA fragments were then stained with SYBR Safe and visualized with UV trans-illuminator. DNA profiles from such markers were scored in comparison with their parents. Plant selection in each generation was dependent on a number of plants that carried the target regions. Step-wise marker assisted selection and phenotyping technique was applied to select, to advance the chosen plants, and to decrease the number of samples in every generation.

### Selection process

In this study, the selection process involved four steps. First is the foreground selection for the MR219 population where the *qDTY*_*2.2*_*, qDTY*_*3.1*_ and *qDTY*_*12.1*_ loci were monitored by RM236, RM520, and RM511 markers, respectively, which are tightly linked with those QTLs [18, 22]. Once the individuals with donor alleles at the peak of the *qDTY* region/s were identified, these individuals were genotyped with an additional three to six markers flanking both sides of the *qDTY* region/s. This second step is also known as recombinant selection and its main purpose is to increase the efficiency of selection by reducing linkage drag [40, 60]. Moreover, the use of flanking markers for recombinant selection also assisted in recovering the important traits of the recipient parent and in minimizing the effects of linkage drag from the *qDTY* donors. In the third step, the individuals which passed the foreground and recombinant selections will undergo phenotypic screening. Here, only individuals with desired plant traits such as appropriate plant height (90 to 120 cm), long grains, and free of diseases were short listed. The first three steps of selection were performed in every generation of development of the PLs. However, only in the BC_1_F_1_ generation, selected individuals were also genotyped with background markers in order to determine the percentage of the recipient parent genome. In the fourth step, plants showing positive interactions between QTLs/QTL combinations and background as evidenced by higher yield under drought were selected.

### Performance of pyramided lines

After genotyping, 198 BC_1_F_3_ (separated between short and medium duration PLs) and 525 BC_1_F_5_ PLs with different *qDTYs* and their combinations were selected based on similarity in morphological characteristics with MR219. These 198 BC_1_F_3_ and 525 BC_1_F_5_ PLs were evaluated together with their recipient and donor parents in the field under lowland RS and NS conditions during the 2013 DS and 2014 DS. Field-based phenotyping trials were conducted at the IRRI farms in lowland transplanted conditions (IRRI, Los Banos, Philippines, 14^0^ N 121^0^E, 21 m above sea level). Lowland refers to field trials conducted under flooded, puddled, and transplanted conditions. In total, six lowland trials (three under RS and three under NS) were conducted using this population. MR219 PLs and MR219 were evaluated in an alpha lattice design with two replications [61] in plot sizes of two rows of 5 m length at 25 cm × 25 cm spacing. Missing hills were replanted with stock seedlings within 10 days of transplanting. A total of 50 plants were maintained in each plot. Inorganic fertilizers (N:P:K) were applied at the rate of 90:30:30 kg ha^−1^. The post emergence herbicide Sofit (pretilachlor 0.3 kg a.i. ha^−1^) was applied four days after transplanting and hand weeding was done for weed control. For the control of stem borers and other insects, Furadan (carbofuran 1 kg a.i. ha^−1^) at five days after transplanting and Cymbush (cypermethrin 1 kg a.i. ha^−1^) at 16 days after transplanting were applied. To control snails, a molluscicide, Bayluscide (niclosamide 0.25 kg a.i. ha^−1^) was also applied to the fields.

For the RS trials, the fields were irrigated to maintain soil moisture at field capacity or above for four weeks after transplanting. RS was imposed four weeks after transplanting by draining water from the field. Perforated PVC pipes were placed at a 100-cm soil depth in four different points in the field. Daily water table depth was measured in the RS trials after stress initiation. Data on daily rainfall, daily maximum and minimum temperature, and relative humidity for the trial period were recorded. The fields were allowed to dry until the soil cracked and the surface was completely dry. When the target level of soil dried and the check varieties as well as 70 % of the entries showed severe leaf rolling, and the water table reached below 100 cm and remained the same for about three weeks, irrigation was introduced through flash flooding. The fields were drained again after 24 h [19] to impose the second cycle of stress. Parching water table was measured from all pipes every day after draining the field until the crop reached 50 % maturity.

In the NS trials, 5 cm water level was maintained in the fields throughout the crop season until draining before harvesting. The NS trials were conducted to obtain the data on the performance of PLs under control condition to select lines combining high yield under NS and considerably good yield under RS conditions.

### Data collection

Data for DTF, PH, and GY were recorded from all trials. DTF was recorded as the number of days from sowing till the day when 50 % of the plants had flowering tillers. PH (in cm) of three plants from each plot was measured at maturity from ground level to the tip of the tallest tiller and averaged for analysis. GY from each plot was harvested at physiological maturity, dried to 14 % moisture content, and weighed. The measured GY was then converted to kg ha^−1^.

### Statistical analysis

Recorded data from the RS and NS trials were compiled separately. Data from each trial were analyzed using CROP STAT v7.2 and PB Tools v1.1.0 softwares (http://bbi.irri.org/products) based on a mixed model that considers replications and blocks within replications as random effect and the genotypes as fixed effect. Trial-wise broad-sense heritability (*H*) for each trait was calculated as:

$$ H = \frac{\sigma_g^2}{\sigma_g^2+{\sigma}_e^2/r} $$

where *σ*_*g*_^2^ is genotypic variance, *σ*_*e*_^2^ is error variance, and *r* is the number of replications.

### Selection of drought-tolerant pyramided lines

BC_1_F_5_ PLs with different *qDTY* combinations that produced more than 900 kg ha^−1^ under RS but yielding similar or more than MR219 under NS were identified during the 2014 DS. These identified PLs were classified as drought tolerant. A total of 16 BC_1_F_5_ PLs were classified as the most promising as they produced more stable and consistent GY across the two dry seasons. The MR219 PLs with single and different combinations of *qDTYs* were categorized into eight classes depending on whether they possessed one (class E, F, and G), two (class B, C, and D) or three (class A) *qDTYs* or none (class X).

### QTL combinations class analysis

The performance of the genotype nested within the QTL class in the block within the replicate is modelled as follows:

$$ {y}_{ijkl}=\mu +{r}_k+b{(r)}_{kl}+{q}_i+g{(q)}_{ij}+{e}_{ijkl} $$

where *μ* is the population mean, *r*_*k*_ is the effect of the *k*^*th*^ replicate, *b*(*r*)_*kl*_ + *q*_*i*_ is the effect of the *l*^*th*^ block within the *k*^*th*^ replicate, *q*_*i*_ is the effect of the *i*^*th*^ QTL, *g*(*q*)_*ij*_ is the effect of the *j*^*th*^ genotype nested within the *i*^*th*^ QTL and *e*_ijkl_ is the error [62]. The effects of QTL and genotypes within QTL are considered fixed while the replicate and blocks within replicate effects are considered random.

## Availability of data and material

All the data from which conclusions of this research are drawn are present in Tables 1, 2, 3 and 4 and Figs. 1, 2, 3, 4 and 5.

**Table 2:** Means for days to flowering (DTF), plant height (PH) and grain yield (GY) of MR219 PLs as compared to MR219 under lowland reproductive stage drought stress and non-stress conditions

Season/Year	Stress	Duration	No. of MR219 PLs	DTF			PH (cm)			GY (kg ha^-1^)			
				Mean MR219 PLs	Mean MR219	Trial H	Mean MR219 PLs	Mean MR219	Trial H	Mean MR219 PLs	RYR (%)	Mean MR219	Trial H
DS2013	Non-stress	Short	108	81	89	0.82	90	104	0.54	6040	93	6639	0.53
DS2013	Drought	Short	108	87	-	0.81	60	-	0.27	397	-	0	0.76
DS2013	Non-stress	Medium	90	85	91	0.75	95	91	0.75	6774	86	5917	0.54
DS2013	Drought	Medium	90	92	-	0.88	71	-	0.36	920	-	0	0.87
DS2014	Non-stress	Medium	525	99	100	0.86	87	97	0.7	7120	91	8319	0.68
DS2014	Drought	Medium	525	90	-	0.78	60	-	0.52	672	-	0	0.58

MR219 did not flower under drought stress

Dry season (DS), Days to 50 % flowering (DTF), and plant height (PH, in cm), broad-sense heritability (H), grain yield (GY, in kg ha^−1^) and relative yield reduction in RS compared to NS (RYR, in percentage)

**Table 3:** QTL presence, and grain yield (GY) of 16 chosen best performers - across two seasons of lowland drought stress and non-stress conditions

Line	Duration in 2013DS	*qDTY*_*12.1*_	*qDTY*_*3.1*_	*qDTY*_*2.2*_	GY (kg ha^−1^)			
					DS2013		DS2014	
					RS	NS	RS	NS
IR 99784-156-137-1-1	Medium	-	√	-	1362	6200	2523	10713
IR 99784-255-7-2-5	Medium	√	√	-	1346	7783	1591	8383
IR 99784-188-202-1-2	Medium	-	√	√	939	9431	1562	8369
IR 99784-188-202-1-1	Medium	-	√	√	939	9431	1478	7937
IR 99784-255-68-1-5	Medium	√	√	√	1611	6905	1183	7782
IR 99784-188-202-1-3	Medium	-	√	√	939	9431	1058	6947
IR 99784-255-55-2-5	Medium	√	-	√	2073	8525	1049	7858
IR 99784-188-201-B-1	Medium	√	-	√	977	8610	1042	9975
IR 99784-255-7-2-2	Medium	√	√	-	1346	7783	983	9438
IR 99784-40-1-B-6	Medium	-	-	√	1164	8168	942	8254
IR 99784-11-35-2-2	Short	-	√	√	1300	8633	-	-
IR 99784-255-9-1-3	Medium	√	√	-	888	8174	936	9014
IR 99784-156-87-1-9	Medium	√	√	√	923	5808	930	11672
IR 99784-188-179-1-2	Medium	√	√	-	1459	9107	927	7419
IR 99784-11-8-1-5	Short	-	-	√	756	9573	-	-
IR 99784-255-49-1-1	Medium	√	√	-	2521	5832	903	8152
MR 219		-	-	-	13	5917	0	8322

MR219 did not flower under drought stress

**Table 4:** QTL class mean comparisons for grain yield in kg ha^−1^ under reproductive stage drought stress (stress) and irrigated control (non-stress) in MR219 as the recipient parent in trials conducted during dry season 2013 and 2014

QTL class label	QTL	2013 - short duration		2013 - medium duration		2014 - medium duration	
		NS	RS	NS	RS	NS	RS
A	*qDTY*_*2.2*_ *+ qDTY*_*3.1*_ *+ qDTY*_*12.1*_	4600 ba	475.55 c	6799 ab	642 b	7706 d	442 b
B	*qDTY*_*12.1*_ *+ qDTY*_*3.1*_	6577 d	179.57 ab	6652 ac	1072 d	7532 cd	794 e
C	*qDTY*_*12.1*_ *+ qDTY*_*2.2*_	4518 ba	376.55 c	7633 b	761 bc	7364 ac	698 d
D	*qDTY*_*2.2*_ *+ qDTY*_*3.1*_	6867 d	444.98 c	7158 bc	1112 d	6843 b	571 c
E	*qDTY*_*12.1*_	5935 c	373.46 cb	6229 a	654 b	6967 b	301 a
F	*qDTY*_*3.1*_	6366 dc	582.25 c	6488 a	890 c	7374 ac	568 c
G	*qDTY*_*2.2*_	4148 a	514.48 c	6760 ab	1104 d	7079 ba	669 dc
X (MR 219)	NO QTL	6713 bdc	0 a	5917 ab	13 a	8321 bcd	0 ab
F- value		7.48	3.27	2	11.76	9.45	19.39
p-value		<0.0001	0.0043	0.07	<0.0001	<0.0001	<0.0001

Means followed by the same letter are not significantly different

## Abbreviations

DTF: days to flowering
*qDTYs*: drought yield QTLs
DS: dry season
GY: grain yield
H: heritability
IRRI: International Rice Research Institute
KDML 105: Khao Dawk Mali 105
MAB: marker assisted breeding
MMAL: molecular marker application
NS: non-stress
PBGB: plant breeding, genetics, and biotechnology
PH: plant height
PLs: pyramided lines
QTLs: quantitative trait loci
RS: reproductive stage drought stress
SSR: simple sequence repeat
